# Career choices for ophthalmology made by newly qualified doctors in the United Kingdom, 1974–2005

**DOI:** 10.1186/1471-2415-8-3

**Published:** 2008-03-04

**Authors:** Trevor W Lambert, Michael J Goldacre, Anthony J Bron

**Affiliations:** 1 UK Medical Careers Research Group, Department of Public Health, Oxford University, Oxford, UK; 2 Nuffield Laboratory of Ophthalmology, University of Oxford, Oxford, UK

## Abstract

**Background:**

The paper aims to report trends in career choices for ophthalmology among UK medical graduates.

**Methods:**

Postal questionnaire surveys were undertaken of qualifiers from all UK medical schools in nine qualification years since 1974. Data were analysed by univariate cross-tabulation. The significance of comparisons between groups of doctors were calculated by the use of chi-squared tests and adjusted residuals.

**Results:**

Ophthalmology was the first choice of long term career for 2.3% of men and 1.5% of women one year after qualification; 2.0% of men and 1.4% of women three years after; and 1.8% of men and 1.2% of women at five years. Comparing early choices with eventual destinations, 64% who chose ophthalmology in year one, 84% in year three, and 92% in year five eventually practised in the specialty. The concordance between year one choice and eventual destination was higher for ophthalmology than for most other specialties. 'Enthusiasm for and commitment to the specialty' was the most important single factor in influencing career choice. The prospect of good working hours and conditions was also an important influence: it influenced career choice a great deal for a higher percentage of those who chose ophthalmology (66% in the third year) than those who made other surgical choices (23%).

**Conclusion:**

Those choosing ophthalmology show a high level of commitment to it. Their commitment is strengthened by the prospect of attractive hours and working conditions. Many doctors who become ophthalmologists have already made their choice by the end of their first post-qualification year.

## Background

We have undertaken surveys of all medical qualifiers from all medical schools in the United Kingdom in selected years since 1974. Our aims are to document the career choices, career progression and career destinations of the doctors; and to report on their views about various aspects of working in medicine [1-4]. Our findings have been published as they have emerged from individual surveys [1-4] but have not, until now, been compiled into a single summary paper for each specialty.

Doctors' career choices, and views about their jobs and training, provide insights which can assist with workforce planning. Information about the certainty of choices, whether and when doctors change their minds about their career intentions, and the relationship between choice and eventual career destinations, are all helpful to those who design training programmes and plan workforce supply.

In addition to data on career choice and certainty of choice, we present data on factors affecting career choices for ophthalmology. There are few published UK data on this topic; a US study in 2003 [5] and a Canadian study in 2004 [6] provide the most recent comparative information. In the US, lifestyle considerations were important to those who chose general ophthalmology careers, while those who chose specific subspecialties within ophthalmology were more likely to do so because of the perceived prestige and personal marketability which such skills would provide. The Canadian ophthalmologists cited intellectual stimulation, flexibility of employment, and earning potential as important factors in forming their career choice.

In this paper we summarise and expand on our findings, from all cohorts studied by us, about early career choices for ophthalmology.

## Methods

Surveys have been undertaken by the UK Medical Careers Research Group of the UK medical graduates of 1974, 1977, 1980, 1983, 1993, 1996, 1999, 2000 and 2002. Our methods have been described in detail elsewhere [2,3]. In brief, towards the end of the first and third years after graduation, and at longer time intervals after that, we send postal questionnaires to all medical graduates from each UK medical school. Up to four reminders are sent to non-respondents. In 1974 the graduates of all medical schools in England, Wales and Scotland were surveyed. From the cohort of 1977 onwards, the surveys covered the whole of the UK including Northern Ireland.

The doctors who were mailed in the first survey of each cohort comprised the whole cohort as it was at the time of qualification. Subsequent surveys of a cohort excluded only those doctors who previously had indicated that they did not wish to participate, or who were untraceable, or who were known to have died. For the initial survey, addresses were obtained from the doctors' registration with the General Medical Council. For follow-up surveys, we used the addresses supplied by the doctors at each previous survey and/or those identified from the most recent Medical Registers and Medical Directories.

We ask each doctor *"Have you made up your mind about your choice of long-term career?*", asking them to choose a response from '*definitely*,' '*probably*' or '*not really*.' We ask them to specify their choice in their own words and to be as general or specific as they wish. If they have more than one choice, we ask them to list up to three in order of preference and, if applicable, to indicate that choices are of equal preference. Additionally, we invited those who graduated in 1993, 1996, 1999, 2000, and 2002 to signify which factors, from a list of 11 possible factors specified in the questionnaire, had influenced their choice of career '*a great deal*,' '*a little*' or '*not at all*.' For this paper, we selected choices for ophthalmology. We compare these with choices for the other surgical specialties, combined (Appendix), and for non-surgical specialities combined.

The data were analysed by univariate cross-tabulation and the significance of comparisons between groups of doctors were calculated by the use of chi-squared tests and adjusted residuals.

Ethical approval for the UKMCRG cohort studies has been obtained through the Central Office for Research Ethics Committees (COREC), following referral to the Brighton Mid Sussex and East Sussex local research ethics committee.

## Results

### Response rate

The survey questionnaires were sent to a total of 33,141 UK doctors covering all nine cohorts in the pre-registration year. A total of 24,621 (74.3%) replied. Three years after qualification a second survey questionnaire was sent to the same doctors and 23,468 (70.8%) replied. Five years after qualification all except the 1983 and 2002 cohorts were surveyed a third time and 17,689 out of 24,870 (71.1%) replied. Combining the surveys, 71.5% (13164/18407) of men and 77.8% (11457/14734) of women responded in the pre-registration year (χ^2^_1 _= 166.7, p < 0.001), with similar differences in response rates by gender in years three and five.

### Ophthalmology as doctors' choice of long-term career, as expressed in the early years after qualification

Ophthalmology was the first choice of long term career for 2.3% of men and 1.5% of women one year after qualification; 2.0% of men and 1.4% of women three years after; and 1.8% of men and 1.2% of women at five years. The percentage differences by gender were all statistically significant, with p < 0.001 using a standard chi-squared test. The percentage of doctors who specified ophthalmology as their first choice of long term career, in responding at the end of the pre-registration year, increased a little between the cohorts of 1974–83 and those of 1993–2002 (Table 1). Tests for linear trend across the cohorts showed a significant increasing trend among men for the choice of ophthalmology, but not among women (see footnotes to Table 1 for test results).

**Table 1:** Percentage (and numbers) of respondents who specified Ophthalmology as their first choice of eventual career at one (1974–2002), three (1974–2000) and five (1974–1980, 1993 & 1996) years after graduation

	Percentages (numbers) choosing Ophthalmology								
	
	Year One			Year Three			Year Five		
			
Cohort	Men	Women	Total	Men	Women	Total	Men	Women	Total
			
1974	0.9 (13)	0.8 (4)	0.9 (17)	1.3 (14)	1.4 (6)	1.3 (20)	1.2 (15)	1.2 (6)	1.2 (21)
1977	1.4 (25)	0.9 (8)	1.3 (33)	1.5 (24)	1.4 (11)	1.5 (35)	1.5 (26)	1.2 (11)	1.4 (37)
1980	2.0 (36)	2.5 (25)	2.1 (61)	1.7 (31)	1.4 (14)	1.6 (45)	1.4 (25)	1.0 (10)	1.3 (35)
1983	2.2 (42)	1.0 (12)	1.7 (54)	2.2 (40)	0.8 (10)	1.6 (50)	N/A	N/A	N/A
1993	3.5 (45)	2.2 (29)	2.8 (74)	1.9 (27)	1.8 (25)	1.9 (52)	2.0 (27)	1.7 (23)	1.8 (50)
1996	1.9 (27)	1.3 (20)	1.6 (47)	1.7 (21)	1.5 (22)	1.6 (43)	2.1 (25)	1.0 (14)	1.5 (39)
1999	2.7 (31)	1.7 (27)	2.1 (58)	2.4 (26)	1.4 (20)	1.8 (46)	2.4 (28)	1.2 (18)	1.7 (46)
2000	3.1 (40)	1.4 (23)	2.1 (63)	3.0 (40)	1.7 (28)	2.3 (68)	2.4 (29)	1.2 (18)	1.7 (47)
2002	3.6 (39)	1.6 (27)	2.4 (66)	2.9 (31)	0.8 (14)	1.6 (45)	N/A	N/A	N/A
Total	2.3 (298)	1.5 (175)	1.9 (473)	2.0 (254)	1.4 (150)	1.7 (404)	1.8 (175)	1.2 (100)	1.6 (275)

Numbers of respondents were 13164 (Men), 11457 (Women) and 24621 (Total) in Year One; 12420 (Men), 11048 (Women) and 23468 (Total) in Year Three; 9559 (Men), 8128 (women) and 17687 (Total) in Year Five. Chi-square test for linear trend – year 1; men χ^2^_1 _= 27.7 (p < 0.001); women χ^2^_1 _= 0.9 (p = 0.35); total χ^2^_1 _= 14.6 (p < 0.001) – year three; men 10.6 (p = 0.001); women 0.4 (p = 0.85); total 3.5 (p = 0.062) – year five; men 10.5 (p = 0.001); women 0.02 (p = 0.90); total 4.1 (p = 0.043).

Considering all choices, second and third as well as first, ophthalmology was a career choice for 3.4% of men and 2.3% of women in year one, 2.4% of men and 1.6% of women in year three, and 2.0% of men and 1.4% of women in year five (Table 2), differences by gender which were also statistically significant with p < 0.001. Similar trends over time to those in the year 1 choices were observed, with a significant increase for men but not for women; see footnotes to Table 2 for test results.

**Table 2:** Percentage (and numbers) of respondents who specified Ophthalmology as their first, second or third choice of eventual career at one (1974–2002), three (1974–2000) and five (1974–1980, 1993 & 1996) years after graduation

	Percentages (numbers) choosing Ophthalmology								
	
	Year One			Year Three			Year Five		
			
Cohort	Men	Women	Total	Men	Women	Total	Men	Women	Total
			
1974	1.7 (24)	0.9 (5)	1.5 (29)	2.1 (22)	1.4 (6)	1.9 (28)	1.5 (19)	1.8 (9)	1.6 (28)
1977	2.8 (50)	2.1 (18)	2.6 (68)	1.7 (27)	1.7 (13)	1.7 (40)	1.8 (31)	1.4 (12)	1.7 (43)
1980	2.9 (53)	3.6 (36)	3.1 (89)	2.3 (42)	1.7 (17)	2.1 (59)	1.7 (29)	1.3 (13)	1.5 (42)
1983	3.3 (62)	1.8 (23)	2.7 (85)	2.5 (45)	1.1 (13)	1.9 (58)	N/A	N/A	N/A
1993	4.6 (60)	2.4 (32)	3.5 (92)	2.2 (31)	1.9 (26)	2.1 (57)	2.2 (29)	1.7 (23)	1.9 (52)
1996	3.0 (42)	2.4 (37)	2.7 (79)	1.8 (23)	1.7 (25)	1.8 (48)	2.1 (25)	1.1 (15)	1.6 (40)
1999	4.0 (46)	2.1 (33)	2.9 (79)	2.9 (32)	1.7 (24)	2.2 (56)	2.4 (28)	1.3 (19)	1.8 (47)
2000	4.2 (55)	2.0 (33)	3.0 (88)	3.4 (45)	1.9 (31)	2.6 (76)	2.6 (31)	1.3 (19)	1.8 (50)
2002	4.9 (53)	2.4 (41)	3.4 (94)	3.3 (35)	1.0 (17)	1.9 (52)	N/A	N/A	N/A
Total	3.4 (445)	2.3 (258)	2.9 (703)	2.4 (302)	1.6 (172)	2.0 (474)	2.0 (192)	1.4 (110)	1.7 (302)

Numbers of respondents were 13164 (Men), 11457 (Women) and 24621 (Total) in Year One; 12420 (Men), 11048 (Women) and 23468 (Total) in Year Three; 9561 (Men), 8128 (women) and 17689 (Total) in Year Five. Chi-square test for linear trend – year 1; men χ^2^_1 _= 22.9 (p < 0.001); women χ^2^_1 _= 0.04 (p = 0.84); total χ^2^_1 _= 7.7 (p = 0.005) – year three; men 7.0 (p = 0.008); women 0.01 (p = 0.91); total 1.5 (p = 0.23) – year five; men 5.8 (p = 0.02); women 0.5 (p = 0.48); total 0.7 (p = 0.40).

### Certainty of choice

Doctors who specified, at the end of the first year, that ophthalmology was their first choice of career were as definite about their choice (36% were definite) as doctors who chose other branches of surgery (36%); and aspiring ophthalmologists and other surgeons were more definite about their choice of future career than those who expressed a first choice for other clinical careers (26%; Table 3). By year three, 68% who chose ophthalmology said that they were certain about their choice, compared with, respectively, 44% who chose other surgical careers and 47% who chose non-surgical careers; and by year five 80% were certain compared with 72% and 65%. There was some change over time in certainty of choice: for example, of those who chose ophthalmology in year one, 28% of the qualifiers of 1974–1983 and 40% of those of 1993–2002 said that they were definite about their choice (p = 0.01 using the Mann-Whitney U test to compare all 3 categories). The corresponding percentages for year three choices were 61% in the cohorts of 1974–1983 and 73% in the cohorts of 1993–2002 (p = 0.03); by year five the difference was small at 78% compared with 81% (p = 0.62).

**Table 3:** Comparing those who chose Ophthalmology with those who chose other careers, percentages and numbers of doctors who specified whether they had definitely, probably or not really made up their minds about their first choice of long-term career, at one (1974–2002), three (1974–2000) and five (1974–1980, 1993 & 1996) years after graduation

	First choice of long term career		
	
Definiteness of choice	Ophthalmology	Other Surgery	Other Careers
	
	Year One		
	
Definitely	36.0 (169)	36.0 (1403)	26.3 (5257)
Probably	45.4 (213)	47.2 (1841)	49.1 (9833)
Not really	18.6 (87)	16.8 (653)	24.6 (4936)
Total	100.0 (469)	100.0 (3897)	100.0 (20026)

	Year Three		
	
Definitely	68.1 (273)	43.5 (1237)	46.5 (9164)
Probably	23.7 (95)	47.4 (1348)	40.6 (8012)
Not really	8.2 (33)	9.1 (259)	12.9 (2540)

Total	100.0 (401)	100.0 (2844)	100.0 (19716)

	Year Five		
	
Definitely	79.6 (218)	72.0 (1330)	64.8 (9888)
Probably	17.5 (48)	24.4 (450)	29.0 (4422)
Not really	2.9 (8)	3.7 (68)	6.3 (961)

Total	100.0 (274)	100.0 (1848)	100.0 (15271)

The numbers of respondents who did not to answer this question were 4 (Year One), 3 (Year Three) and 1 (Year Five) of those choosing Ophthalmology; 42 (Year One), 13 (Year Three) and 12 (Year Five) of those choosing Other surgical specialties, and 183 (Year One), 491 (Year Three) and 281 (Year Five) of those choosing Other Careers.

Chi-square tests on the percentage making a definite choice: Year One; ophthalmology vs. other surgery χ^2^_1 _= 0.0, p = 1.0, ophthalmology vs. other careers χ^2^_1 _= 22.0, p < 0.001 – Year Three; ophthalmology vs. other surgery χ^2^_1 _= 84.4, p < 0.001, ophthalmology vs. other careers χ^2^_1 _= 72.8, p < 0.001 – Year Five ophthalmology vs. other surgery χ^2^_1 _= 6.6, p = 0.01, ophthalmology vs. other careers χ^2^_1 _= 25.3, p < 0.001

### Differences between medical schools

Using logistic regression with the year of qualification, sex and medical school as predictors of the choice of ophthalmology, there was little evident variation between medical schools in the level of choice for ophthalmology (χ^2^_22 _= 37.1, 36.1, 18.7, in years one, three and five; p = 0.02, 0.36, 0.66, respectively). Detailed information on the numbers and percentages of students from each medical school who chose ophthalmology are shown in the Table 4.

**Table 4:** Percentages (and numbers) of doctors from each medical school who specified Ophthalmology as first choice of career at one (1974–2002), three (1974–2000) and five (1974–1980, 1993 & 1996) years after graduation

Clinical Medical School	Percentages (Numbers) choosing Ophthalmology		
	
	Year One	Year Three	Year Five
Birmingham	1.9 (21)	1.4 (15)	1.3 (11)
Bristol	2.0 (18)	1.4 (12)	1.5 (10)
Cambridge	4.0 (21)	2.7 (14)	1.6 (6)
Leeds	1.6 (15)	1.2 (11)	1.5 (11)
Leicester	1.8 (11)	1.5 (9)	1.3 (5)
Liverpool	1.5 (14)	1.1 (10)	0.8 (6)
Manchester	2.4 (40)	2.0 (31)	1.9 (22)
Newcastle	1.6 (15)	1.6 (15)	1.3 (9)
Nottingham	1.6 (12)	2.0 (15)	1.7 (9)
Oxford	3.4 (21)	3.1 (18)	2.8 (12)
Sheffield	1.4 (14)	0.9 (8)	1.2 (6)
Southampton	3.4 (24)	2.6 (18)	2.4 (12)
Imperial College	1.8 (32)	1.9 (31)	1.6 (20)
King's College	2.2 (44)	2.0 (38)	1.8 (26)
Queen Mary & Westfield	1.6 (23)	2.2 (28)	1.9 (19)
St George's	2.3 (16)	3.0 (19)	2.7 (13)
University College London	1.5 (31)	1.7 (32)	1.4 (21)
Aberdeen	1.5 (12)	1.0 (8)	0.8 (5)
Dundee	2.7 (20)	2.2 (15)	1.9 (10)
Edinburgh	1.3 (16)	1.0 (12)	1.3 (12)
Glasgow	1.1 (16)	1.0 (14)	1.1 (11)
Wales	1.8 (18)	1.6 (14)	1.1 (8)
Belfast	2.2 (19)	1.6 (14)	1.5 (9)

Total	1.9 (473)	1.7 (401)	1.6 (275)

Numbers of respondents were 24611 in Year One, 23377 in Year Three and 17686 in Year Five. The medical school is unknown for 10 Year One respondents, 91 Year Three respondents and 1 Year Five respondent: these respondents are excluded.

### Early choices and eventual destinations

In Table 5, we compare early career preferences with eventual career destinations. The results show career destinations ten years after qualification for the cohorts of 1974–1993 and seven years after for the cohort of 1996. They show early career preferences subdivided into those doctors who said that ophthalmology was their first and only choice, and those who gave ophthalmology as a tied first choice with another specialty.

**Table 5:** Percentages and numbers of doctors who originally chose Ophthalmology and were working in Ophthalmology seven (1996 cohort) and ten (1974, 1977, 1983 and 1993 cohorts) years after qualification

	**Specialty at Year Seven/Ten**			
	
	**Percentages (Numbers)**			
	
Original Choice	**Ophthalmology**	**Other Surgery**	**Other Careers**	**All Specialty Destinations**
	
Ophthalmology in Year One (untied 1^st ^choice)	63.8 (111)	4.6 (8)	31.6 (55)	100 (174)
Ophthalmology in Year One (tied 1^st ^choice)	21.1 (4)	5.3 (1)	73.7 (14)	100 (19)
Other surgery in Year One	1.1 (20)	46.2 (822)	52.7 (937)	100 (1779)
Other career in Year One	0.4 (41)	0.9 (90)	98.7 (9834)	100 (9965)
*Total (All Year One Choices)*	*1.5 (176)*	*7.7 (921)*	*90.8 (10840)*	*100 (11937)*
				
Ophthalmology in Year Three (untied 1^st ^choice)	84.0 (147)	0.6 (1)	15.4 (27)	100 (175)
Ophthalmology in Year Three (tied 1^st ^choice)	25.0 (2)	0.0 (0)	75.0 (6)	100 (8)
Other surgery in Year Three	0.3 (3)	68.4 (809)	31.3 (370)	100 (1182)
Other career in Year Three	0.1 (12)	0.6 (57)	99.3 (9781)	100 (9850)
*Total (All Year Three Choices)*	*1.5 (164)*	*7.7(867)*	*90.8 (10184)*	*100 (11215)*
				
Ophthalmology in Year Five (untied 1^st ^choice)	92.0 (127)	0.0 (0)	8.0 (11)	100 (138)
Ophthalmology in Year Five (tied 1^st ^choice)	- (0)	- (0)	- (0)	- (0)
Other surgery in Year Five	0.0 (0)	82.9 (731)	17.1 (151)	100 (882)
Other career in Year Five	0.1 (5)	0.6 (44)	99.4 (7786)	100 (7835)
*Total (All Year Five Choices)*	*1.5 (132)*	*8.8 (775)*	*89.8 (7948)*	*100 (8855)*

There was a high level of concordance between early career choices for ophthalmology and eventual careers in the specialty. Of those who gave the specialty as their sole first choice, 64% who chose it in year one were working in it eventually, as were 84% who chose it in year three, and 92% who chose it in year five. As a comparison, the corresponding figures for concordance between year one, three and five choices, and eventual career destination, were 46%, 68% and 83% for the other surgical specialties; 37%, 58% and 80% for the hospital medical specialties; and 74%, 82% and 88% for general practice. Tied first choices for ophthalmology with other specialties were much less highly predictive of eventual destinations (Table 5).

Those who chose ophthalmology as their untied first choice in year one and did not eventually work in it numbered 63 (Table 5); the most common career destinations for them were general practice (20 doctors), not in employment (18), other surgical specialties (6), medical specialties (6) and anaesthetics (3).

### Factors that influenced career choice for ophthalmology

We asked the doctors in the cohorts of 1993 to 2002 to rate eleven factors according to whether they had influenced their choice of eventual career *a great deal*,*a little*, or *not at all *(Table 6).

**Table 6:** Percentages of doctors whose first choice of long-term career was for ophthalmology and other careers, who specified each factor as influencing their choice of long-term career a great deal: graduates of 1993–2002

	**First choice of long-term career**			
	
**Factor influencing career choice 'a great deal'**	**Ophthal mology**	**Other surgery**	**Other careers excluding GP**	**GP**
				
	% (n)	% (n)	% (n)	% (n)
**Year One**				
Domestic circumstances	15.5 (46)	**7.9**(189)	13.7 (986)	**37.7**(1319)
Hours/working conditions	60.7 (181)	**17.4**(414)	**35.2**(2546)	**73.7**(2597)
Future financial prospects	29.4 (87)	**19.0**(453)	**10.1**(728)	**15.7**(552)
Career & promotion prospects	28.6 (85)	26.8 (639)	25.0 (1803)	**16.5**(580)
Self-appraisal of own skills/aptitudes	42.1 (125)	**50.9 **(1212)	49.7 (3582)	46.3 (1625)
Advice from others	29.4 (87)	**16.2**(383)	**16.6**(1199)	**16.3**(570)
Experience of chosen subject as a student	47.5 (141)	49.7 (1187)	46.1 (3336)	**36.9**(1296)
^†^A particular teacher/department	28.5 (84)	**38.3 **(914)	29.6 (2135)	**13.4**(469)
Inclinations before medical school	11.1 (33)	16.8 (401)	13.7 (992)	14.8 (519)
Experience of jobs so far	33.4 (99)	**62.1**(1483)	**50.8**(3678)	**48.4**(1703)
Enthusiasm/commitment: what I really want to do	69.5 (205)	75.1 (1789)	65.0 (4661)	**59.2 **(2070)
				
**Year Three**				
Domestic circumstances	20.6 (26)	**10.5 **(103)	17.6 (718)	**48.8**(1073)
Hours/working conditions	66.4 (85)	**23.0**(226)	**39.5**(1619)	**81.7**(1815)
Future financial prospects	25.2 (32)	19.6 (193)	**8.9**(364)	22.7 (502)
Career & promotion prospects	16.5 (21)	26.4 (260)	24.4 (998)	22.8 (502)
Self-appraisal of own skills/aptitudes	47.7 (61)	48.2 (471)	51.9 (2121)	50.0 (1102)
Advice from others	24.6 (31)	17.9 (175)	16.5 (671)	16.6 (366)
Experience of chosen subject as a student	40.6 (52)	29.0 (286)	**26.3**(1076)	**20.8**(459)
^†^A particular teacher/department	25.0 (21)	38.7 (244)	30.3 (797)	**11.1**(159)
Inclinations before medical school	19.5 (25)	14.9 (146)	12.2 (501)	14.8 (327)
Experience of jobs so far	48.0 (61)	**74.4**(733)	**68.5**(2806)	54.2 (1198)
Enthusiasm/commitment: what I really want to do	78.4 (98)	71.5 (702)	**65.3 **(2663)	**56.5**(1245)
				
**Year Five**				
Domestic circumstances	37.8 (65)	**18.5**(215)	29.5 (1562)	**64.4**(2153)
Hours/working conditions	70.5 (122)	**24.9**(290)	**45.9**(2432)	**85.4**(2858)
Future financial prospects	21.4 (37)	17.1 (200)	**8.1**(432)	17.3 (580)
Career & promotion prospects	20.8 (36)	27.1 (316)	25.0 (1330)	19.8 (664)
Self-appraisal of own skills/aptitudes	51.5 (88)	57.4 (671)	57.2 (3045)	51.3 (1719)
Advice from others	20.3 (35)	17.4 (203)	14.2 (754)	**10.7**(359)
Experience of chosen subject as a student	30.2 (52)	26.2 (307)	22.1 (1175)	**17.1**(573)
^†^A particular teacher/department	12.8 (11)	**37.2**(241)	24.8 (664)	**5.2 **(89)
Inclinations before medical school	8.7 (15)	12.2 (143)	9.9 (529)	12.6 (423)
Experience of jobs so far	52.3 (90)	**76.3**(893)	**68.2**(3635)	52.1 (1749)
Enthusiasm/commitment: what I really want to do	79.8 (138)	84.0 (983)	75.0 (3992)	**59.9**(2013)
^††^Financial circumstances whilst training	2.0 (1)	5.2 (14)	5.0 (72)	7.3 (62)
^††^Organisation of training programme	16.0 (8)	22.3 (60)	29.9 (428)	27.2 (232)
^††^Inability to secure qualifications	6.3 (3)	1.5 (4)	2.1 (29)	2.5 (21)

Year One uses data from the 1993, 1996, 1999, 2000 and 2002 cohorts, Year Three from the 1993, 1996 and 2002 cohorts, and Year Five from the 1993, 1996, 1999 and 2000 cohorts.

^†^This statement was not presented to graduates of 1996 in their third year after graduating, or the graduates of 1993 and 1996 in their fifth year after graduating.

^†† ^These three statements were only presented to the 1993 graduates in their fifth year after graduating.

Excluding these statements, the smallest number of respondents to any statement in Years One, Three and Five respectively was for those choosing ophthalmology 295, 126 and 171, for other surgical specialties 2366, 978 and 1165, for other specialties excluding GP 7167, 4068 and 5287 and for GP 3495, 2199 and 3343.

In each row, where percentages for the three specialty choices other than ophthalmology differ significantly from the corresponding percentage for ophthalmology, a **bold underlined** percentage indicates p < 0.001 and **bold alone **indicates p < 0.01.

Two factors were rated by the majority of doctors who chose ophthalmology as having a great deal of influence – *Enthusiasm/commitment: what I really want to do *and *Hours and working conditions. Enthusiasm/commitment *influenced career choice 'a great deal' for 70% of those who chose ophthalmology at year one, 78% at year three and 80% at year five. These are similar to the percentages for those who chose other surgical specialties and slightly higher than for those who chose non-surgical careers (though only significantly so in year three). The doctors' perception of anticipated hours and working conditions influenced career choice 'a great deal' for a significantly higher percentage of doctors who chose ophthalmology (61% in year one) than in other surgical specialties (17%) or non-surgical specialties other than general practice (35%), with only those choosing general practice scoring higher (74%). This influence of hours and working conditions on career choice was maintained in years three and five.

The doctors' *domestic circumstances*, probably often related to their views about the importance to them of working hours, grew in influence over the years and in each year influenced the career choice of a significantly higher percentage of those who chose ophthalmology than those who chose other surgical specialties. This level of influence was similar to that for doctors who chose non-surgical specialist careers but it was important to a much lower percentage of aspiring ophthalmologists than of general practitioners.

*Future financial prospects *were a greater influence on choices for ophthalmology than for other specialties in year one. However, this influence declined slightly with time and by year five there was no significant difference between ophthalmology and other surgical careers or general practice. However, future financial prospects remained more important for ophthalmologists than for doctors in the non-surgical specialties.

*Advice from others *was significantly more influential on career choice for ophthalmologists than other groups in year one; in years three and five this influence decreased.

The initial (year one) influence of *Experience of chosen subject as a student *was similar for ophthalmology and other surgical specialties and other specialist careers, but was lower for general practice, a difference which was sustained in years three and five.

*Career and promotion prospects *were a greater influence on year one career choices for ophthalmologists than for general practitioners; differences in later years were small.

*Experience of jobs so far *grew in influence for ophthalmologists over the years but remained substantially less influential than for other areas of specialist practice in each year studied.

Influence of *a particular teacher or department *was less important for aspiring ophthalmologists than for those who chose other areas of surgery, but more important than for those who chose general practice.

*Self-appraisal of own skills and aptitudes *was less significant as an influence in year one for ophthalmologists than for those who chose other areas of surgery.

*Inclinations before medical school *was not a particularly important influence on career choice.

Statistically significant differences in percentages are highlighted in Table 6.

## Discussion

### Strengths of the study

Our studies have created a large, national, longstanding, longitudinal database of information about doctors' career choices which is certainly unique in the UK, and probably unique in the world. The prospective nature of the studies is a particular strength – we have captured the views and aspirations of the doctors, as they were at the time, in the early years after qualification; and can compare their early views and plans with what they subsequently did. Thus the studies are not subject to either recall bias or weakened by the doctors' lack of recollection about early intentions. Ophthalmology is a specific and easily identifiable career choice in the data, and we are therefore confident that our results reliably reflect the respondents' career intentions.

### Weaknesses

Although the response rate of over 70% is high in the context of postal questionnaire surveys, there remains the possibility of non-responder bias in ways that are hard to identify. In common with many other questionnaire-based studies, we have a higher response rate from women than from men. We have no evidence about any other differences between respondents and non-respondents.

Our questionnaires are fairly brief in order to encourage a high response rate; consequently we do not ask detailed questions about the motivations which may influence career choice.

### General discussion

As a numerically small specialty within clinical practice, ophthalmology only needs and will only attract a small percentage of medical graduates. Our results show a modest increase over time in the popularity of ophthalmology as a career choice. Since the early 1990s there has been a sharp decline in the popularity of general practice, particularly among men, and an increase in the popularity of the surgical specialties; the increase in choices for ophthalmology may in part reflect these broader trends. Although a higher percentage of men than women expressed a choice for ophthalmology, in terms of career choice the specialty is less 'male dominated' than surgery as a whole.

Two factors could influence future trends in doctors' choice of ophthalmology as a career. Firstly, it is evident that perceptions about good working conditions have influenced career choices for ophthalmology. As long hours of work decline in the UK in all clinical specialties, ophthalmology may lose some of its competitive advantage among the surgical specialties. Secondly, according to a November 2006 statement from the Royal College of Ophthalmologists [7], there is currently a larger number of trainees completing specialist training in ophthalmology than the number of available consultant posts in the specialty in the UK. However, this mismatch may be rectified in the future as the treatability of common retinal conditions such as age-related macular degeneration is recognised and addressed [8]. Furthermore, because a high proportion of workload in ophthalmology is concerned with care of elderly patients, the increasingly large number of elderly in the general population will tend to increase workload in the specialty. New treatments, together with the demographic effect of an ageing population fuelling additional demand for existing treatments, will necessitate the creation of more consultant posts in the specialty.

Compared with most other specialties, a particularly high percentage of doctors who specify that they want a career in ophthalmology, in the early years after qualification, eventually practice in the specialty. This is relevant to current debate in the UK about when doctors should commit themselves to their long-term specialty choice. Current policies in the UK have moved towards earlier commitment than in the past, through *Modernising Medical Careers*[9]. However, there are also some concerns that early commitment may reduce junior doctors' flexibility in trying out a range of specialties at the junior level before making a final choice [10]. Our evidence shows that many doctors who become ophthalmologists have already made their choice by the end of their first post-qualification year.

## Conclusion

Our findings from the study of factors that influenced career choice indicate that, particularly compared with other hospital specialties, choices for ophthalmology are influenced, for most of those who choose it, by the importance they attach to attractive working hours and a good working environment; with financial prospects being also regarded as a more important factor by those choosing ophthalmology than those choosing other careers. However, the importance of the experience of jobs undertaken was not as great among ophthalmologists as among other specialists. As a finding in the first year after qualification, this might be explained by a relative lack of exposure to ophthalmology in the early clinical years, but we could not explain why this finding persisted in three and five. Notwithstanding this, enthusiasm and commitment to the specialty was high among those who chose it, and was similar to that in other surgical specialties. These findings were similar to those in the US and Canadian studies [5,6]. Once young medical graduates have decided that they want to go into ophthalmology, they tend to be strongly committed to the specialty.

## Competing interests

The author(s) declare that they have no competing interests.

## Authors' contributions

TWL and MJG planned and designed the surveys. TWL analysed the data and performed the statistical analysis. TWL and MJG wrote the first draft in collaboration with AJB. All authors contributed to further drafts and approved the final manuscript.

## Pre-publication history

The pre-publication history for this paper can be accessed here:


